# Synthesis and Characterization of Ionic Graft Copolymers: Introduction and In Vitro Release of Antibacterial Drug by Anion Exchange

**DOI:** 10.3390/polym12092159

**Published:** 2020-09-22

**Authors:** Katarzyna Niesyto, Dorota Neugebauer

**Affiliations:** Department of Physical Chemistry and Technology of Polymers, Faculty of Chemistry, Silesian University of Technology, 44-100 Gliwice, Poland; katarzyna.niesyto@polsl.pl

**Keywords:** pharmaceutical anion, ion exchange, graft copolymers, choline, drug delivery

## Abstract

Amphiphilic graft copolymers based on [2-(methacryloyloxy)ethyl]trimethyl- ammonium chloride (TMAMA) were obtained for the delivery of pharmaceutical ionic drugs, such as *p-*aminosalicylate (PAS) and clavunate (CLV) anions. The side chains were attached by *grafting from* a multifunctional macroinitiator via atom transfer radical polymerization (ATRP) to get polymers with different grafting degrees and ionic content. The self-assembling ability, confirmed by determining the critical micelle concentration (CMC) through interfacial tension (IFT) with the use of goniometry, was reduced after ion exchange (CMC twice higher than for chloride anions contained copolymers 0.005–0.026 mg/mL). Similarly, the hydrophilicity level (adjusted by the content of ionic fraction) evaluated by the water contact angle (WCA) of the polymer film surfaces was decreased with the increase of trimethylammonium units (68°–44°) and after introduction of pharmaceutical anions. The exchange of Cl^−^ onto PAS^−^ and CLV^−^ in the polymer matrix was yielded at 31%–64% and 79%–100%, respectively. The exchange onto phosphate anions to release the drug was carried out (PAS: 20%–42%, 3.1–8.8 μg/mL; CLV: 25%–73%, 11–31 μg/mL from 1 mg of drug conjugates). Because of the bacteriostatic activity of PAS and the support of the action of the antibiotics by CLV, the designed water-soluble systems could be alternatives for the treatment of bacterial infections, including pneumonia and tuberculosis.

## 1. Introduction

Traditional medicine does not use the full potential of therapeutics when the drug dose at destination is reduced by premature decay, or its concentration is too high and damaging for cells. Hence, the pharmaceutical industry is looking for better solutions in the production of medicines. The drug delivery systems (DDS) based on polymer carriers seem to solve the drug distribution problem with conventional medicines [1,2,3,4,5]. These systems are commonly used to improve bioavailability and increase therapeutic efficacy [1,6,7,8]. Besides, the carrier enables the controlled release of the drug [8,9,10,11,12] by maintaining its constant concentration and avoiding exceeding the toxicity threshold. The polymeric carrier has to be biocompatible and nontoxic to the healthy cells [9,13,14,15,16]. Among the nonlinear polymer graft topology, where the side chains are attached to a main chain [17,18,19,20,21], it is convenient to adjust the physico- and biochemical properties by grafting degree, the length of backbone and grafts. The well-defined graft copolymers can be obtained by the *grafting from* reaction with the use of multifunctional macroinitiators [22,23] and direct formation of polymeric ionic liquids (PILs) as the side chains [24,25].

PILs consist of repeating groups containing ionic pairs in the polymer chain [26,27,28]. They have the unique properties of ionic liquids and exhibit increased mechanical strength and durability [27,29,30,31]. Ionic liquids (ILs) are mostly defined as green solvents, but nowadays the studies have indicated that some of them can be toxic [32,33]. One of the lowest toxicities has been reported for cholinium-based ionic liquids, which have been applied to prepare biocompatible gels [34]. The choline is a water soluble organic chemical compound that has a quaternary ammonium group, usually it is in the form of a chloride [35,36]. This molecule supports biological functions [34,37,38] as a precursor of acetylcholine [39]. The presence of an ionic group in the cholinium unit gives the opportunity for the ion exchange reaction, which in the case of polymerized ionic liquids can be advantageous for incorporation of pharmaceutical ions, i.e., sulfacetamid, salicylate [40,41,42]. There are also reports of other types of PILs, based on imidazolium and pyridinium transporting naproxen anions [43] or guanidinium with ampicillin anions [44].

In this work we report on new PIL systems based on the grafted polymers, which are capable of the delivery of ionic drugs introduced by anion exchange in suitable media imitating body fluids in the human body. The main part of the study was focused on designing graft copolymers, in which the polymerizable ionic liquid [2-(methacryloyloxy)ethyl]trimethylammonium chloride in various ratios with methyl methacrylate were contained in the side chains P(MMA-*co*-TMAMA). The *grafting from* strategy required the preparation of multifunctional macroinitiators, that is copolymers of methyl methacrylate and 2-(2-bromoisobutyryloxy)ethyl methacrylate P(MMA-*co-*BIEM) with two different contents of bromoester initiating groups to get in the next step the copolymers with various grafting degrees. The ion exchange reaction was carried out with sodium *p*-aminosalicylate (NaPAS) or potassium clavunate (KCLV), which are a pharmaceutics used as the second-line antituberculosis drug or in combination with a broad-spectrum activity antibiotic, in the treatment of infections of the respiratory tract. The studies on drug release via anion exchange supported by phosphate anions contained in buffer solution were performed to evaluate the physicochemical effectiveness of systems in the delivery of pharmaceutical anions with antibacterial activity.

## 2. Materials and Methods

Methyl methacrylate (MMA) and 2-(hydroxyethyl) methacrylate (HEMA) (both Alfa Aesar, Warsaw, Poland), were dried, whereas [2-(methacryloyloxy)ethyl]trimethylammonium chloride (TMAMA, 80% aq. solution, Sigma-Aldrich, Poznan, Poland) was concentrated to a constant weight by water evaporation. Anisole (99%, Alfa Aesar, Warsaw, Poland) was desiccated using 4Å molecular sieves (Chempure, Piekary Ślaskie, Poland). Copper(I) bromide and chloride (CuBr and CuCl; both Fluka, 98%, Steinheim, Germany) were purified by stirring in glacial acetic acid, followed by filtration and washing with ethanol and diethyl ether, then dried under vacuum; 2,2′-Bipyridine (bpy), 4,4′-dinonyl-2,2′-dipyridyl (dNbpy, 97%), tetrahydrofuran (THF), pyridine (99%), α-bromoisobutyrate bromide (BIBB, 98%), ethyl 2-bromoisobutyrate (EBiB), potassium clavunate (KCLV), and sodium p-aminosalicylate (NaPAS) were used as received (all Sigma Aldrich, Poznan, Poland).

### 2.1. Synthesis of Multifunctional Macroinitiators

The synthesis procedure includes ATRP to obtain copolymers of 2-hydroxyethyl methacrylate and methyl methacrylate, and then esterification of the hydroxyl groups to introduce the bromoester initiating group. Details are presented in the Supplementary Materials (Table S1, Figures S1–S2).

### 2.2. Synthesis of Graft Copolymers Bearing Cl^−^ (Example for G2)

Comonomers TMAMA (1.80 g, 8.66 mmol) and MMA (2.74 mL, 25.90 mmol), methanol (2 mL), THF (1 mL), bpy (54.14 mg, 0.35 mmol) and macroinitiator Ia (196.50 mg, including 0.35 mmol of initiating sites) were placed into a Schlenk flask and degassed by two freeze-pump-thaw cycles. The initial sample was taken and the CuCl catalyst (25.83 mg, 0.17 mmol) was introduced to the mixture. The reaction was carried out for 1 h, then the first part of the mixture (3 mL) was taken out and then stopped by exposing to air. The reaction with the rest of the mixture was continued for another 1 h, and then stopped. The polymer was twice precipitated in chloroform-diethyl ether mixture, and then dried.

### 2.3. Ionic Exchange for the Introduction of Pharmaceutical Anions (Example for G2)

The copolymer G2 (20 mg, including 0.03 mmol of TMAMA units) was dissolved in methanol (1 mL). Then the pharmaceutical salt, NaPAS (7.90 mg, 0.03 mmol) or KCLV (5.56 mg, 0.03 mmol) was added to keep the reaction for 48 h. The products containing PAS (26.2 mg) or CLV (19.66 mg) were obtained after drying under reduced pressure. Yields: 92% and 98%, respectively.

### 2.4. Drug Release of Pharmaceutical Anions

The grafted copolymers with exchanged pharmaceutical anions (1.0 mg) were dissolved in 1 mL of phosphate buffered saline (PBS) solution (pH = 7.4). Then, a dialysis membrane bag (MWCO = 3.5 kDa) filled out by the solution (1 mL) was introduced into a glass vial with 45 mL of PBS and stirred at 37 °C. During the drug release, buffer samples (2.5 mL) were taken at appropriate intervals and analyzed on a UV−Vis spectrophotometer, measuring absorbance at λ = 265 (PAS^−^) or 257 nm (CLV^−^). The amount of drug in the release medium (*d*) was calculated with the use of following formulas:

(1) $$x=\frac{y-b}{a}$$

(2) $$d=\frac{x}{c}*100\%$$

where: *a* is the value of the slope of the calibration curve, *b* is the intercept of the calibration curve, *x* is a value of the concentration on the basis of the calibration curve equation, *y* is a value of absorbance at the determined wavelength for PAS^−^ (λ = 265 nm) and CLV^−^ (λ = 257 nm).

### 2.5. Characterization

^1^H-NMR spectra were registered by a UNITY/NOVA (Varian, Mulgrave, Victoria, Australia) spectrometer operating at 300 MHz. The measurements were performed for the samples in deuterated dimethyl sulfoxide (DMSO) with tetramethylsilane (TMS) as an internal standard. The monomer conversion was determined by gas chromatograph 6850 Network GC System (Agilent Technologies, Santa Clara, CA, USA) using acetone as the solvent. Molecular weight (M_n_) and dispersity index (Ð) were evaluated by size exclusion chromatography (SEC). The measurements for samples of macroinitiators and their precursors were provided in THF line (1100 Agilent 1260 Infinity with differential refractometer MDS RI detector, Agilent Technologies, Santa Clara, CA, USA) at 40 °C with the flow rate of 0.8 mL/min using precolumn guard (5 mm × 7.5 mm) and column PLGel 5 μm MIXED-C 300 (7.5 mm × 300 mm). The calculations were based on polystyrene standards (580–300,000 g/mol). In the case of graft copolymers the SEC measurements were performed in DMF with addition of 10 mM LiBr (Chromatograph Ultimate 3000 with differential refractometer RefractoMax 521 detector, Thermo Fisher Scientific, Waltham, MA, USA) at 50 °C with a flow of rate 0.25 mL/min using precolumn TSKgel Guard SuperMPHZ-H 6um (4.6 mm × 2 cm) and two columns TSKgel SuperMultiporeHZ-H 6 μm (4.6 mm × 15 cm). These calculations were based on poly(ethylene oxide) (PEO) standards (982–969,000 g/mol). Fourier-transform infrared spectroscopy (FT-IR) was conducted with Spectrum Two 1000 FT-IR Infrared Spectrometer with attenuated total reflection (ATR) (Perkin Elmer, Waltham, MA, USA). The critical micelle concentration (CMC) was determined by the measuring interfacial tension (IFT) with the pendant drop method using goniometer (OCA 15EC, DataPhysics, Filderstadt, Germany). The polymer concentration in aqueous solution was ranged in 5 × 10^−4^–0.3 mg/mL. SCA20_U software was used for data collecting and processing. This module software also enabled the measurement of water contact angles (WCA) of water dropped on polymer film. The solution of polymer dissolved in methanol (0.3 mg/mL) was applied to properly prepared and degreased glass plates by spin coating. The sessile drop method was used to drop 4 μL of water. The hydrodynamic diameters (D_h_) of particles and polydispersity index (PDI) were measured by dynamic light scattering (DLS) using a Zetasizer Nano-S90 (Malvern Technologies, Malvern, UK). Samples placed in poly(methyl methacrylate) (PMMA) cells after dilution with a solvent (1.0 mg/mL) were put in the thermostatted cell compartment of the instrument at 25 °C. Each measurement was repeated three times, to create an average value. During drug release, samples taken in appropriate times intervals were analyzed by ultraviolet−visible light spectroscopy in PMMA cells (UV−Vis, spectrometer Evolution 300, Thermo Fisher Scientific, Waltham, MA, USA) to determine the drug content (DC) and the amount of released pharmaceutical anions (PAS^−^ or CLV^−^).

## 3. Results

### 3.1. Synthesis and Characterization of Grafted Copolymers with PIL Side Chains

**Figure 1:**
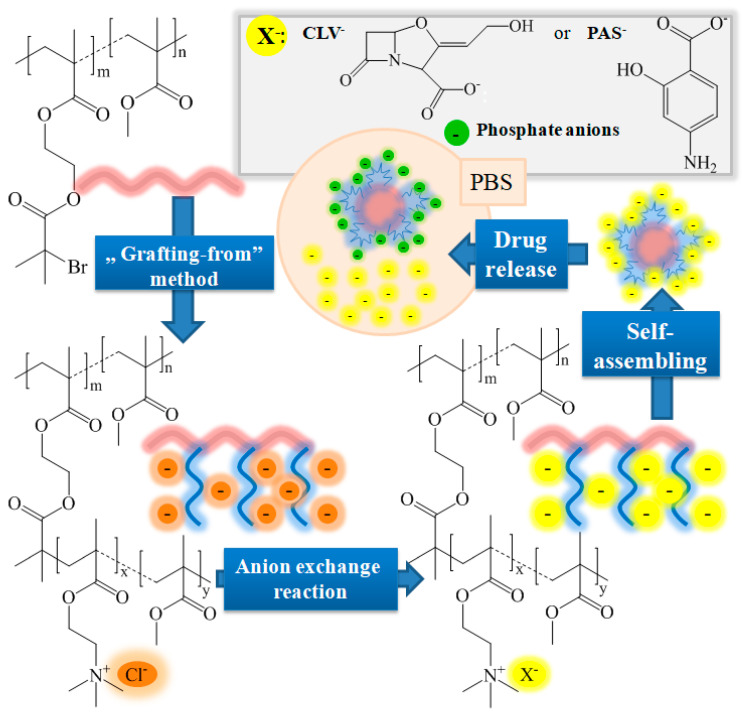
Schematic route for systems based on grafted copolymers with ionic side chains carrying pharmaceutical anions.

**Figure 2:**
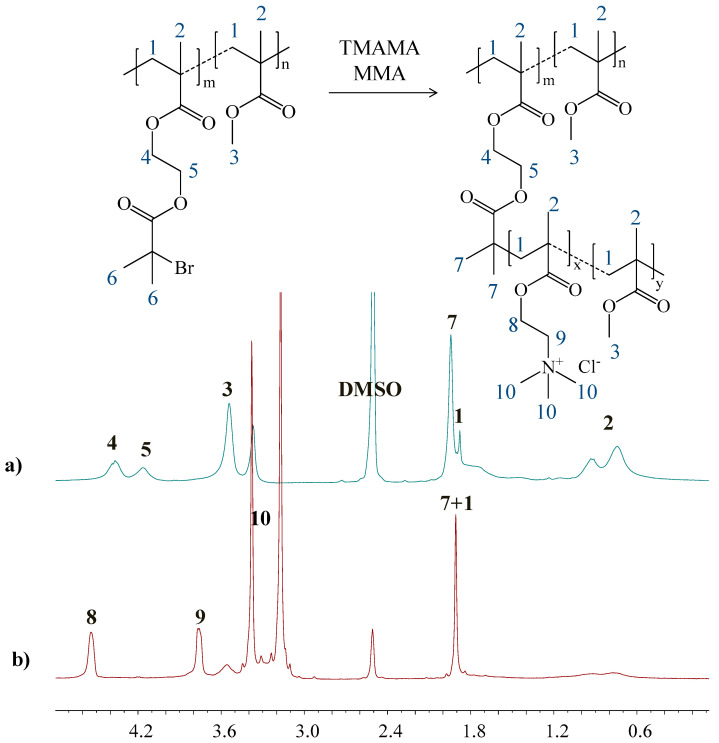
^1^H NMR spectra of (**a**) macroinitiator Ia, and (**b**) grafted copolymer G2.

The grafted copolymers P(MMA-*co*-(BIEM-*graft-*(TMAMA-*co-*MMA))) varying with content of TMAMA units (25% and 50%) and grafting degree (DG = 26% and 46%) were obtained by ATRP catalyzed with CuCl/bpy complex in THF/MeOH at 40 °C (Figure 1). The parameter of grafting degree was adjusted by using a multifunctional macroinitiator (MI) Ia or IIa (Table 1) with different contents of initiating units (DP_BIEM_) in the copolymerization of TMAMA and MMA. The ^1^H NMR spectrum of graft copolymer in comparison to that of its macroinitiator (Figure 2) shows a new signal **10** between 3.4 and 3.0 ppm assigned to 9H in trimethylammonium groups. The formation of side chains was also confirmed by the appearance of two signals **8** and **9** coming from the –CH_2_–O– (3.86–3.66 ppm) and –CH_2_–N^+^ (4.63–4.44 ppm) groups in TMAMA units, respectively. Additionally, the signals of methylene and methyl protons (**1** at 1.9 ppm and **2** ranged in 0.6–1.2 ppm) became more intensive because they are also contained in the polymethacrylate side chains. The conversions of TMAMA presented in Table 2 were calculated from the ^1^H NMR analysis for the reaction mixture by estimating integration of signals from unreacted comonomers TMAMA (6.19–6.07 ppm) in relation to the constant intensity of pyrene signal (8.26–8.18 ppm) used as the internal standard (Figure S3).

### 3.2. Ionic Exchange with NaPAS and KCLV

The exchange reaction of chloride anions in TMAMA units distributed statistically along the side chains was applied to introduce pharmaceutics in the anionic form. This reaction was based on mixing of the appropriate grafted copolymer and the selected bioactive compounds, that is *p-*aminosalicylate sodium salt (NaPAS) or clavunate potassium salt (KCLV), per 48 h at room temperature (Figure 1). PAS as a second-line antituberculosis drug, which belongs to the group of bacteriostatic drugs, is applied as an adjunct to therapy with other antituberculosis drugs, e.g., streptomycin. CLV is β-lactam drug with a penicillin-like structure, which itself does not have clinically significant antibacterial activity, but in combination with an β-lactam antibiotics, e.g., amoxicillin, it inactivates bacterial β-lactamases and thus prevents the breakdown of the antibiotic. The exchange reaction with pharmaceutical salts enables the formation of carrier-drug ionic conjugates as alternative drug delivery systems mostly for the treatment of respiratory tract infections, such as chronic bronchitis, bacterial sinusitis, acute otitis media, community acquired pneumonia and tuberculosis.

The amount of introduced drug in a polymer matrix was verified by drug content (DC, Table 3), which was determined with the use of UV−Vis spectra for systems containing PAS^−^ or CLV^−^. Dependently on the chemical nature of the pharmaceutical substance, the DC in the polymer matrix with the same hydrophilic-hydrophobic balance differed, indicating 31%–64% for PAS and twice as high for CLV (66%–100%), which could also be related to lower steric hindrance of the latter.

### 3.3. Amphiphilic Properties and Wettability

**Figure 3:**
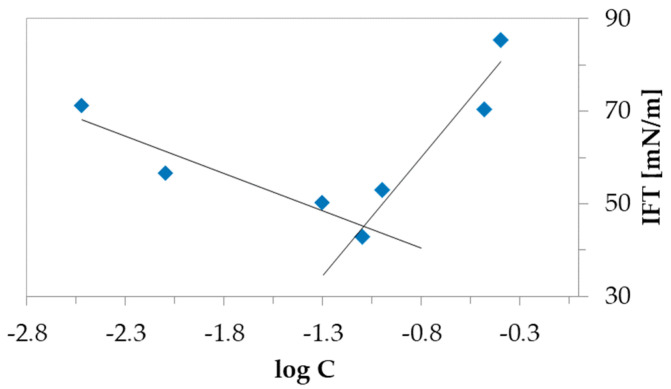
Variation of the surface tension with the logarithm of the concentration of grafted copolymer G2 in aqueous solution at 25 °C.

Because of the amphiphilic character of the obtained graft copolymers with the ionic nature of the side chains (hydrophilic content 10–44 mol.%) the self-assembling superstructures could be formed in aqueous solution. This ability was determined by the CMC value for each polymer system by interfacial tension (IFT), which was convenient for evaluating amphiphilic properties by separating into the interface due to lower surface tension at critical concentration. The goniometry method was used to measure the IFT of aqueous solutions with various concentrations of the grafted copolymers and both types of drug conjugates (c = 5 × 10^−4^–0.3 mg/mL). The IFT of measured samples vs. logC was plotted, where the cross-over point was determined as the CMC value (Figure 3). The CMC data summarized in Table 4 shows that the arrangement of TMAMA units in the polymer have the crucial impact of creating micellar structures. It was noticed that the increase in the chain length resulted in an increase in the CMC value (G1 vs. G2, G3 vs. G4, G5 vs. G6, G7 vs. G8). The highest CMC was observed for samples G7 and G8 (0.020–0.026 mg/mL), which are characterized with both a dense distribution of the side chains and ionic groups among the side chains. Similar to the basic graft polymers with chloride anions, their modified analogs bearing PAS or CLV anions were also self-assembled in aqueous solution (Table 4). In each case the ion exchange with pharmaceutic anions led to a double increase in the CMC value (i.e., G1: 0.01 mg/mL vs G1_PAS: 0.025 mg/mL and G1_CLV: 0.021 mg/mL) maintaining the same correlations as the above-described. This means that the micelles are formed at higher concentrations than in the case of graft polymers with chloride anions as the effect of the hydrophilic nature of the pharmaceutical anions (Cl^−^ < PAS^−^, CLV^−^) which shift the hydrophilic−hydrophobic balance improving solubility of the systems in aqueous solution.

**Figure 4:**
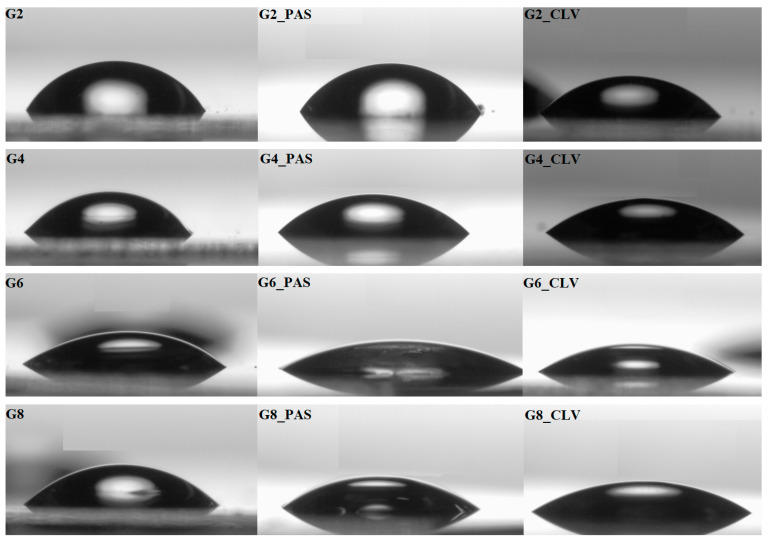
Representative screen shots of the goniometry measured samples.

The use of goniometer was also advantageous for the WCA measurements by sessile water drop method to quantity the wettability of the surface of polymer films made by spin coating. The WCA values presented in Table 4 demonstrate dependence on the grafting density, which supported higher values for low DG (G1–G4) than that for higher grafting density systems (G5–G8). The hydrophobicity of polymers was reduced by the increased chain length and the amount of TMAMA units (Cl: 68°–44°). After exchange with pharmaceutical ions the WCA values were reduced for films of ionic drug polymer conjugates showing the improved hydrophilicity of these systems. The changeable wettability is clearly seen in the attached photos of a water drop on the sample surfaces (Figure 4). Furthermore, a similar relationship between hydrophilicity degree and TMAMA content was also observed for conjugate systems (PAS: 66°–30°; CLV: 64°–29°).

**Figure 5:**
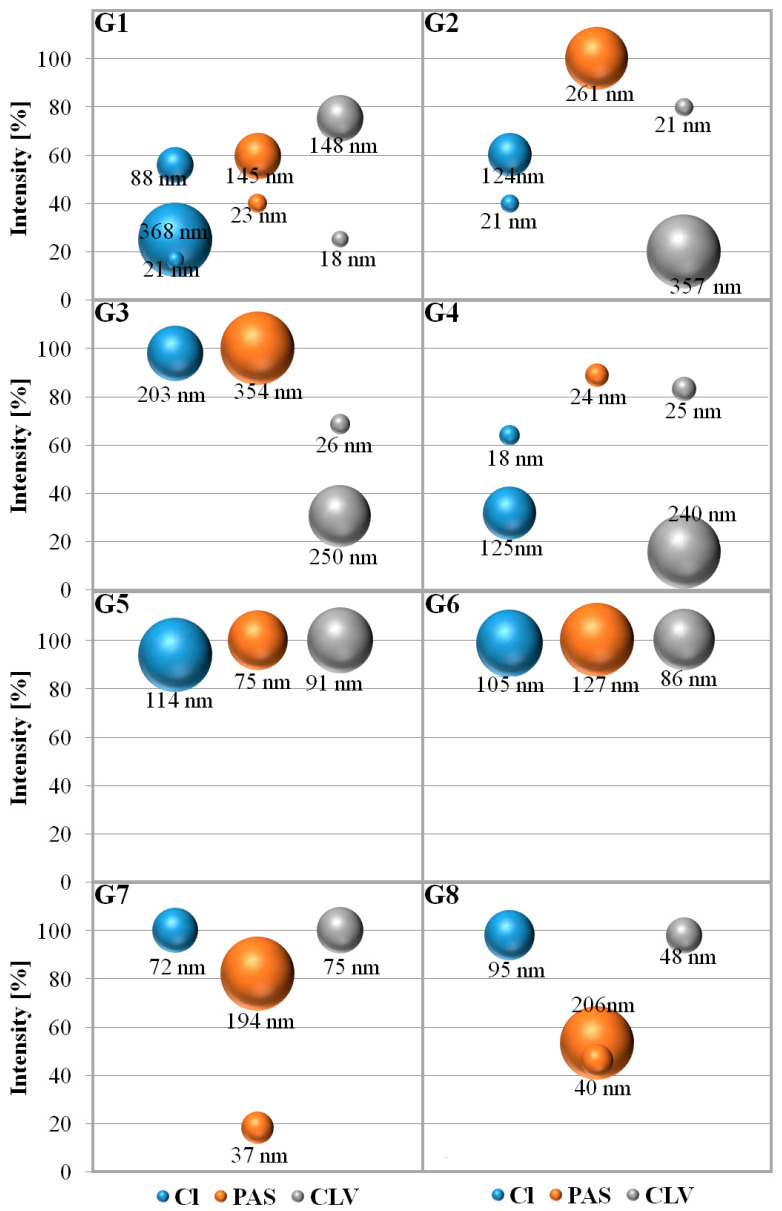
Hydrodynamic diameters (D_h_) of polymer nanoparticles determined by dynamic light scattering (DLS).

Both the chloride containing polymers and those with pharmaceutical anions were analyzed by DLS to determine the hydrodynamic diameters (D_h_) of their particles in water solutions (Figure 5, Figure S6). The graft copolymers with chloride counterions mostly formed one fraction of the superstructures reaching sizes in the range of 72–203 nm (G3, G5–G8), whereas three or two fractions were distinguished in G1, G2 and G4, where the main fraction accounted for ~60% (18–124 nm).

After PAS introduction, the polymer nanoparticles in the prevailing fraction indicated a tendency to increase in size in the range of 24–354 nm, with the exception of G1 and G5 (almost doubly reduced to 145 nm and 75 nm, respectively). Additionally, they were usually characterized by the presence of one fraction, excluding G1, G7 and G8 systems, which existed in two fractions (~20–40 nm and ~140–200 nm as the dominating one). Another relationship was noticed after exchange to CLV^−^, where the particles mostly became smaller (21–148 nm corresponding to the main fraction as 69%–100%). In this series the systems based on higher density graft copolymers G5–G8 were self-assembled to a homogeneous fraction of particles below 100 nm. Comparing all systems, including those containing drugs, the smallest particles were obtained in the case of sample G4, i.e., 18 nm (Cl^−^), 24 nm (PAS^−^), and 25 nm (CLV^−^) as the dominating fraction (64%–90%). The small particles corresponded to small micelle cores, which were provided by G4 due to the highest content of ionic fraction (43%) and the lowest content of hydrophobic fraction (61%) in the group of copolymers with lower density of ionic side chains grafted from shorter hydrophobic main chain in contrast to the series G5–G8. The detailed data with regard to particular nanoparticle fractions, their sizes, and polydispersity index are given in Table S2.

### 3.4. Drug Release

The delivery of PAS and CLV in anionic form is possible in PBS media, because the contained phosphate anions are able to replace PAS^−^ and CLV^−^ in the polymer matrix (Figure 1). The release experiments, which were carried out in pH 7.4 in 37 °C per 48 h, demonstrated the increasing intensity of the drug absorption peak in UV−Vis spectra with the release time of PAS/CLV (Figure S7).

**Figure 6:**
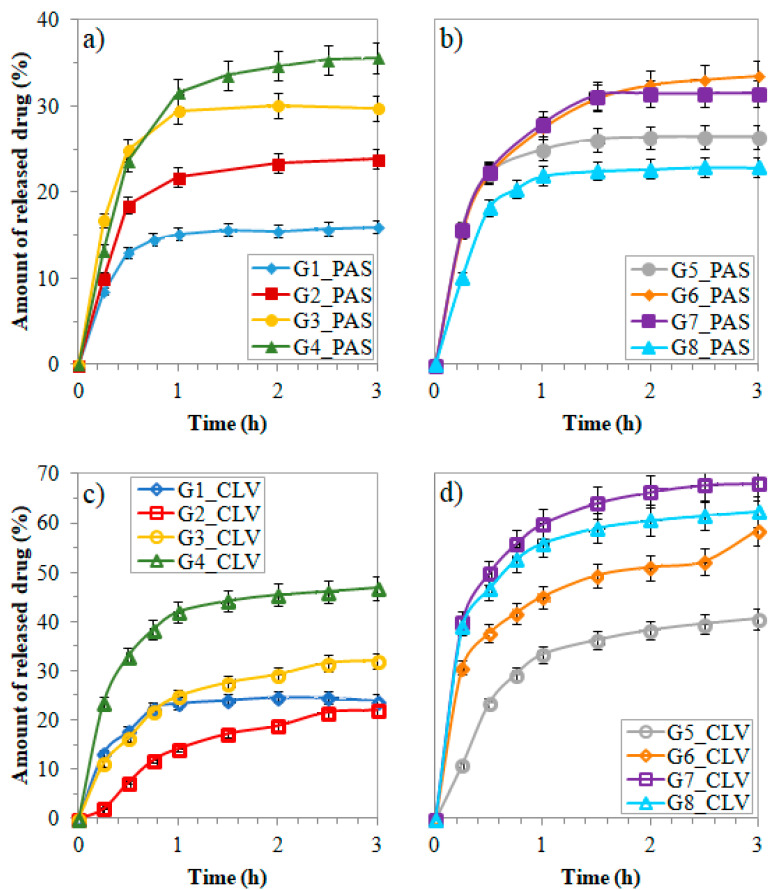
Release profiles of (**a**) and (**b**) PAS, (**c**) and (**d**) CLV anions from systems based on grafted copolymers.

The kinetic profiles in Figure 6 represent an increasing rate of PAS^−^ and CLV^−^ release with the increase in the amounts of ionic groups, especially it was demonstrated by PAS systems of loosely grafted copolymers G1–G4. Effective changes in the amount of released drug were observed for up to three hours (16%–40% of PAS^−^ and 22%–63% of CLV^−^), whereas no significant process progress was noticed above that time up to 48 h (Table 3). Although a low percentage of drug was released by G2_PAS (24%) this system seems to be the most efficient in the PAS delivery (8.8 μg/mL), due to the highest DC of the obtained conjugates (~64%). A similar concentration of released drug was detected for G3_PAS (8.50 μg/mL), i.e., the system with the same grafting degree, but a higher content of ionic units in the side chains. In the case of PAS^−^ based copolymers with low DG, the drug release rate could be regulated by an increase in the side chain length and further improved by the content of ionic moieties in the side chain (G1 < G2 < G3 < G4 in the range of 15%–35% after 3 h, Figure 6a). However, the most efficient drug release was proved for the G7_CLV system (31.1 μg/mL). Similarly, G8_CLV was able to exchange a large amount of the drug (30.4 μg/mL), whereas the DC was almost the lowest in the CLV series (68%).

In comparison with PAS^-^, the CLV^−^ is a more beneficial drug to exchange chloride anions and to be exchanged for phosphate anions in PBS media, especially in the case of the densely grafted copolymers G7–G8 demonstrating the highest release rate (Figure 6d). It means that the interaction of trimethylammonium cations and CLV anions is weaker than with PAS, and additionally the larger number of side chains generates steric hindrance, which is helpful to lose the CLV anions. At the same time, the largest content of ionic fraction at the lower grafting density of G4 provides charge repulsion to increase rigidity of ionic side chains supporting a significantly higher rate of CLV release than for other samples in the series G1–G4 (50% vs. 20%–30% after 3 h, Figure 6c). In turn, the release rate of PAS for system G8 (Figure 6b) with a similar kinetics profile to G2, shows that a double higher grafting degree, combined with high ionic content can be a limiting parameter for drug release due to the relatively strong PAS interactions with the polymer matrix.

## 4. Conclusions

A “grafting from” via ATRP was applied to obtain the ionic graft copolymers based on biocompatible choline, which were modified by exchange with the use of pharmaceutical ions PAS and CLV. These carriers were studied to verify their drug delivery properties by the influence of grafting degree, length and the number of ionic units in the side chains, which can be regulated by proper conditions of reactions. Composition and grafting degree of the copolymers affected the values of CMC to form the self-assembling superstructures at concentrations ranging between 0.005 and 0.026 mg/mL by ionic polymers bearing chloride anions, 0.010 and 0.051 mg/mL for conjugates with PAS ions and 0.012 and 0.040 mg/mL after exchange onto CLV anions. The grafted polymers were beneficial for sufficient drug introduction, but it was more effective for CLV (66%–100%) than in the case of PAS (31%–64%). Similarly, the effect of CLV release by anion exchange in PBS was detected as more valuable, reaching 40%–75% for densely grafted polymers, whereas in the other cases the maximum of released drug provided was in the lower level up to 40%–50% after 48 h. Additionally, it was also noticed for CLV, that in the case of slightly lower DC values (66–89%), these systems supported higher drug release (39%–73% corresponding to 19–31 μg/mL of released CLV^−^). The concentration of PAS was significantly lower (>8.50 μg/mL), which meant that these systems have to be used on a larger scale to deliver therapeutic doses. Our studies indicated that the designed graft copolymers with pharmaceutical counterions seemed to be promising carriers from the physicochemical aspect. In the case of their applications, their rapid action within the three-hour cycle should be effective in the treatment of bacterial infections.

## Figures and Tables

**Table 1:** Data for precursors (I and II), and multifunctional macroinitiators (MI) (Ia and IIa) synthesized by atom transfer radical polymerization (ATRP) and esterification reaction.

No.	HEMA/MMA	DP_HEMA_^a^ (DP_BIEM_)	DP_n_^a^	M_n_^a^ × 10^−3^ (g/mol)	M_n_^b^ × 10^−3^ (g/mol)	Ð ^b^
I	25/75	48	186	20.3	23.4	1.47
Ia		(48)		27.4	22.6	1.37
II	50/50	133	292	33.4	26.5	1.71
IIa		(133)		53.2	33.4	1.63

MMA: Methyl methacrylate, HEMA: 2-(hydroxyethyl) methacrylate; Conditions: I: [HEMA]_0_:[MMA]_0_:[EBiB]_0_:[CuBr]_0_:[dNbpy]_0_ = 150:450:1:1:2, 3 h, II: 300:300:1:1:2, 1 h, anisole 10 vol.% of monomers, 70 °C; bromoesterification of copolymers I, II resulting in Ia and IIa (yield: 100%): pyridine, rt, stirred overnight; ^a^ calculated with the use of monomer conversion by ^1^H NMR, the conversion values in Table S1, DP_n_ is polymerization degree of chain, wherein HEMA before and BIEM after esterification; ^b^ determined by SEC (THF, polystyrene calibration).

**Table 2:** Characteristics of graft copolymers.

No.	MI	Time (h)	n_sc_^a^	DG (mol.%)	X_TMAMA_ (%)	DP_sc_^a^	F_TMAMA_^a^ (mol.%)	M_n_^a^ × 10^−3^ (g/mol)	M_n_^b^ × 10^−3^ (g/mol)	Ð ^b^
G1	Ia	0.5	48	26	9	16	13	114.7	12.5	1.68
G2		1			22	24	21	168.6	17.5	1.9
G3		1			26	31	42	243.6	54.7	1.31
G4		2			30	35	43	273.1	36.3	1.15
G5	IIa	1	133	46	30	29	28	553.9	194.0	1.24
G6		2			48	65	18	1090.5	391.1	1.11
G7		1			22	28	39	583.5	-	-
G8		2			44	48	46	1007.2	-	-

Conditions: G1, G2, G5, G6: [TMAMA]_0_:[MMA]_0_:[MI]_0_:[CuCl]_0_:[bpy]_0_ = 25:75:1:1:2, G3, G4, G7, G8: [TMAMA]_0_:[MMA]_0_:[MI]_0_:[CuCl]_0_:[bpy]_0_ = 50:50:1:1:2, methanol:THF = 2:1 *v/v*, where methanol:TMAMA = 1:1 v/wt, 40 °C; ^a^ determined with ^1^H NMR, where X_TMAMA_ is TMAMA monomer conversion, DP_sc_ is polymerization degree of side chains, F_TMAMA_ is content of TMAMA in side chains, n_sc_ is number of side chains, DG is degree of grafting related to n_sc_ per total DP_n_ of backbone, ^b^ determined with SEC (DMF, PEO calibration).

**Table 3:** Characteristics of polymer bearing *p-*aminosalicylate (PAS)/clavunate (CLV) and evaluation of release effect determined by UV−Vis.

No.	Hydrophilic Fraction ^a^ (mol.%)	Drug Content (DC) (%)		Released Drug after 48 h (%)		Concentration of Released Drug (μg/mL)	
		PAS^−^	CLV^−^	PAS^−^	CLV^−^	PAS^−^	CLV^−^
G1	10	31.2	92.0	18.2	26.1	3.1	13.3
G2	18	63.9	78.6	24.5	25.4	8.8	11.1
G3	37	49.9	98.1	30.6	34.5	8.5	18.8
G4	39	36.2	100	42.4	46.8	7.7	26.7
G5	26	40.2	85.7	30.7	39.1	6.2	18.7
G6	18	37.0	66.3	40.0	61.2	7.4	22.5
G7	36	36.5	88.7	36.5	73.0	6.7	31.1
G8	44	39.7	68.5	28.3	61.8	5.6	30.4

^a^ calculated with the use of ^1^H NMR spectra (Figures S4 and S5).

**Table 4:** Characteristics of graft copolymers in aqueous solution and surface wettability by goniometer method.

No.	CMC ^a^ (mg/mL)			WCA ^b^ (°)		
	Cl^−^	PAS^−^	CLV^−^	Cl^−^	PAS^−^	CLV^−^
G1	0.010	0.025	0.021	68.4	66.4	64.1
G2	0.014	0.032	0.031	61.7	59.0	52.7
G3	0.011	0.019	0.021	60.0	46.3	46.1
G4	0.013	0.028	0.030	56.3	45.7	38.6
G5	0.005	0.010	0.012	53.7	43.5	36.4
G6	0.011	0.036	0.031	44.3	30.3	29.1
G7	0.020	0.044	0.036	48.9	38.6	35.5
G8	0.026	0.051	0.040	46.8	36.5	32.5

CMC: critical micelle concentration, WCA: water contact angle; ^a^ measured by IFT at a concentration 1 mg/mL, ^b^ measured by sessile drop method on the polymer film (polymer solution 0.3 mg/mL spin-coated on glass plate).

## Supplementary Materials

The following are available online at https://www.mdpi.com/2073-4360/12/9/2159/s1, S1: Synthesis of multifunctional macroinitiators, Table S1: Conversion values in synthesis of macroinitiator precursors determined by ^1^H NMR, Figure S1: ^1^H NMR spectra of precursor and multifunctional macroinitiator, Figure S2: FT-IR spectra for precursor and multifunctional macroinitiator, Figure S3: ^1^H NMR spectra of a mixture before starting of reaction, and at the end of copolymerization resulting in grafted copolymer G2, Figure S4: ^1^H NMR spectra of grafted copolymers G2 and G2_PAS, Figure S5: ^1^H NMR spectra of grafted copolymers G5 and CLV G5_CLV, Table S2: Hydrodynamic diameters of nanoparticles determined with DLS, Figure S6: DLS histograms of particles formed by graft copolymers, Figure S7: UV−Vis spectra of released drugs for G2_PAS and G2_CLV systems.
