# Supplementary material for: Synthesis and Characterization of Ionic Graft Copolymers: Introduction and In Vitro Release of Antibacterial Drug by Anion Exchange
Source: Polymers (Basel). 2020 Sep 22;12(9):2159. doi: 10.3390/polym12092159 (PMC7570301; doi:10.3390/polym12092159)
Supplement: Supplementary file 1 [file polymers-12-02159-s001.pdf]

# Supplementary Materials

# Synthesis and characterization of ionic graft copolymers: introduction and *in-vitro* release of antibacterial drug by anion exchange

**Katarzyna Niesyto [1], Dorota Neugebauer [1]***

[1] Department of Physical Chemistry and Technology of Polymers, Faculty of Chemistry, Silesian University of Technology, 44-100 Gliwice, Poland

* Correspondence: Dorota.Neugebauer@polsl.pl

Received: date; Accepted: date; Published: date

## Content:

**S1.** Synthesis of multifunctional macroinitiators P(MMA-*co*-BIEM) (example for Ia).

**Table S1.** Conversion values in synthesis of macroinitiator precursors determined by $^1$H NMR.

**Figure S1**. $^1$H NMR spectra of a) precursor I, and b) multifunctional macroinitiator Ia

**Figure S2**. FT-IR spectra for (a) precursor I, and (b) multifunctional macroinitiator Ia.

**Figure S3**. $^1$H NMR spectra of a mixture a) before starting of reaction, and b) at the end of copolymerization resulting in grafted copolymer G2**.**

**Figure S4.** $^1$H NMR spectra of grafted copolymers a) G2, and b) G2_PAS.

**Figure S5.** $^1$H NMR spectra of grafted copolymers a) G5, and b) G5_CLV.

**Table S2.** Hydrodynamic diameters of nanoparticles determined with DLS.

**Figure S6.** DLS histograms of particles formed by graft copolymers (1 mg/mL aq. solution).

**Figure S7.** UV-Vis spectra of released drug for a) G2_PAS, and b) G2_CLV systems.

S1. Synthesis of multifunctional macroinitiators P(MMA-*co*-BIEM) (example for Ia)

Comonomers HEMA (1.40 mL, 11.50 mmol) and MMA (3.70 mL, 34.50 mmol), anisole (0.50 mL), dNbpy (62.66 mg, 15.30 × $10^{-2}$ mmol) and CuBr (10.99 mg, 7.67 × $10^{-2}$ mmol) were placed into a Schlenk flask and degassed by two freeze–pump–thaw cycles. The initial sample was taken and EBiB initiator (113.77 μL, 7.67 × $10^{-2}$ mmol) was introduced to the mixture. Next, the reaction flask was immersed in an oil bath at 70°C. The reaction was stopped by exposing to air. The reaction mixture diluted in THF was passed through a neutral alumina column to remove copper catalyst, then the polymer was precipitated in diethyl ether and vacuum dried. $^1$H NMR of P(MMA-*co*-HEMA) (Figure S1a) (DMSO-$d_6$, δ, ppm): 5.01–4.72 (1H, –$CH_2$–O**H**), 3.90–3.82 (2H, –C**H**$_2$–OH), 4.17–4.05 (2H, –COO–C**H**$_2$–), 3.56–3.48 3H, –O–C**H**$_3$), 1.94–1.57 (2H, –C**H**$_2$– backbone), 1.4–0.51 (3H, –C**H**$_3$ backbone). FT-IR (Figure S2a) ($cm^{-1}$): 3600–3100 $\nu$(O–H), 3000–2800 $\nu$(C–H), 1750 $\nu$(C=O), 1150 $\nu$(C–O).

The obtained hydroxyl-functionalized polymer **I** (0.70 g, including 0.90 mmol of HEMA units) was dissolved in pyridine (6 mL). Next, the mixture was placed in an ice bath to cool it down to 0 °C. After cooling $\alpha$-bromoisobutyrate bromide (BIBB) (166.21 μL, 1.34 mmol) was added dropwise. The mixture was stirred overnight. Next, the bromoester-functionalized polymer **Ia** was precipitated in cooled water and vacuum dried. $^1$H NMR of P(MMA-*co*-BIEM) (Figure S1b) (DMSO-$d_6$, δ, ppm): 4.47–4.28 (2H, –C**H**$_2$–OOC–C–($CH_3$)$_2$Br), 4.28–4.08 (2H, –COO–C**H**$_2$), 3.74–3.44 (3H, –O–C**H**$_3$), 2.02–1.91 (6H, –(C**H**$_3$)$_2$Br initiating moiety), 1.94–1.57 (2H, –C**H**$_2$– backbone), 1.4–0.51 (3H, –C**H**$_3$ backbone). FT-IR (Figure S2b) ($cm^{-1}$): 3000-2800 $\nu$(C–H), 1750 $\nu$(C=O), 1150 $\nu$(C–O).

**Table S1.** Conversion values in synthesis of macroinitiator precursors determined by $^1$H NMR.

| | HEMA conversion | MMA conversion | total conversion |
|---|---|---|---|
| I | 32 | 31 | 31 |
| II | 44 | 53 | 49 |

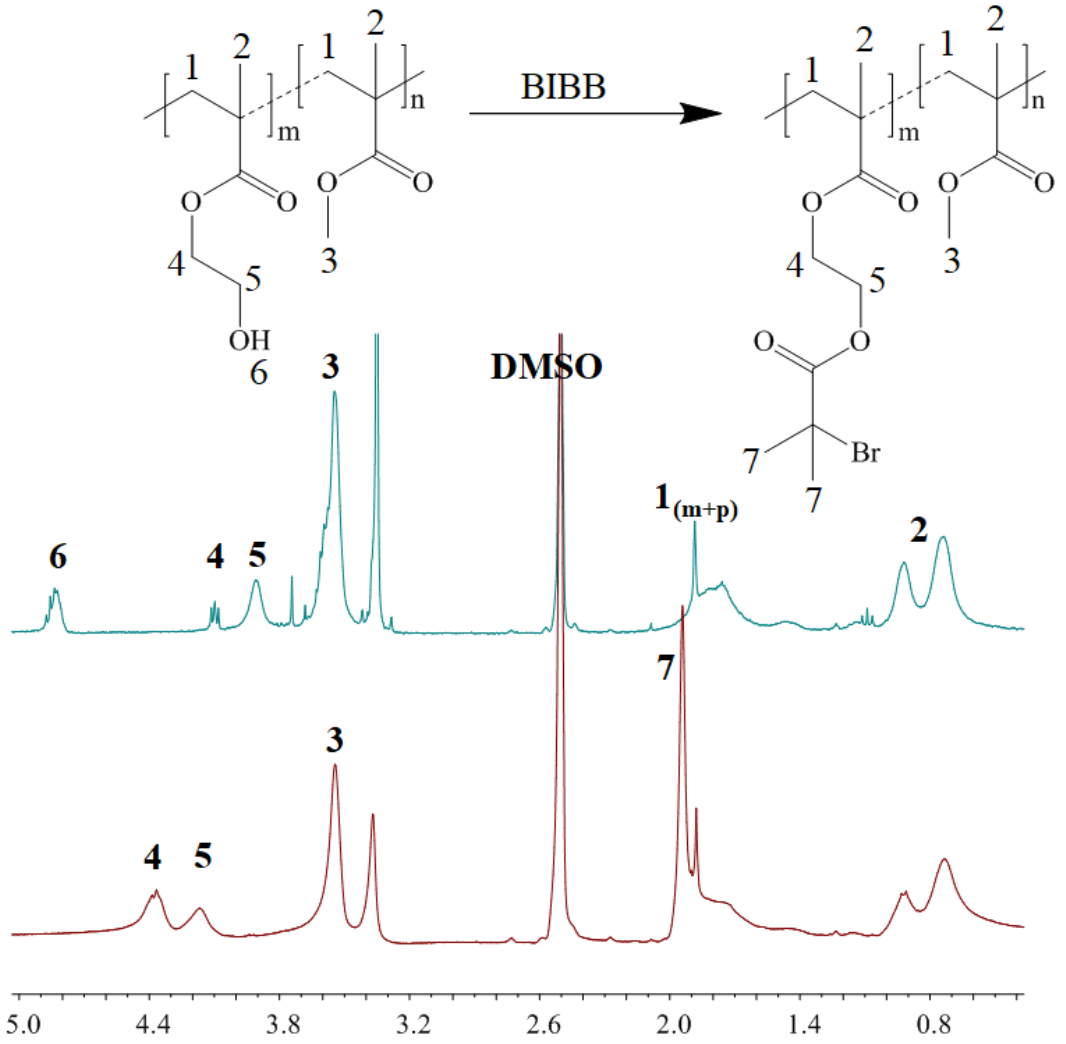


**Figure S1**. $^{1}$H NMR spectra of a) precursor I, and b) multifunctional macroinitiator Ia**.**

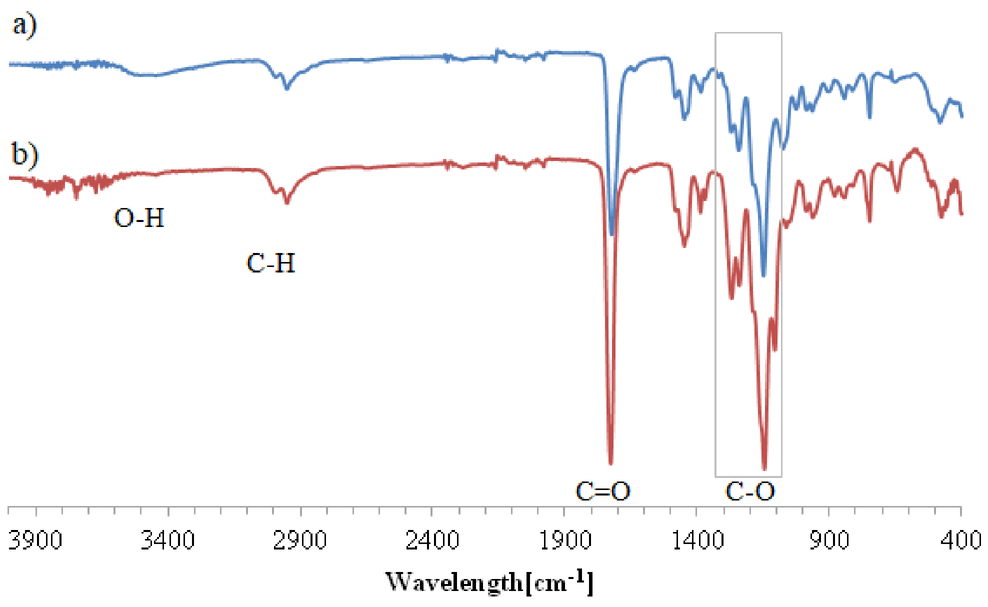


**Figure S2**. FT-IR spectra for (a) precursor **I**, and (b) multifunctional macroinitiator **Ia**.

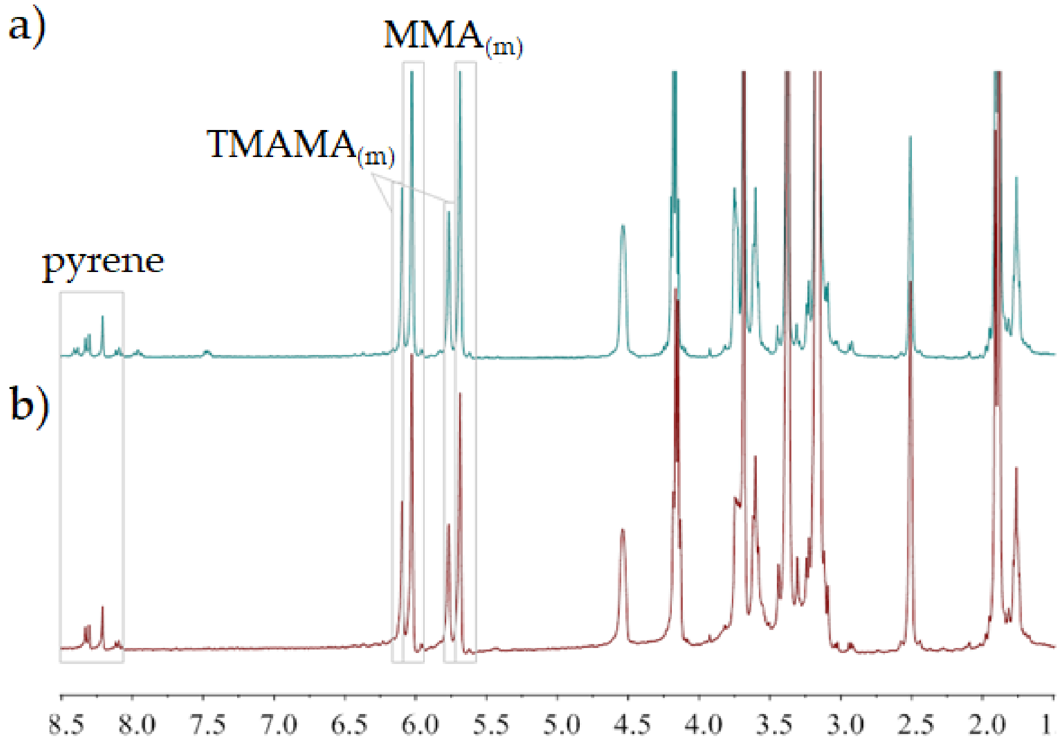


**Figure S3**. $^1$H NMR spectra of a mixture a) before starting of reaction, and b) at the end of copolymerization resulting in grafted copolymer G2**.**

$^1$H NMR (DMSO-d$_6$, δ, ppm): 4.63-4.43 (2H, -C**H$_2$**-O-), 4.47-4.28 (2H, -C**H$_2$**-OOC-C-(CH$_3$)$_2$Br), 4.28-4.08 (2H, -COO-C**H$_2$**), 3.86-3.65 (2H, -C**H$_2$**-N$^+$), 3.65-3.47 (3H, -O-C**H$_3$**), 3.42-3.01 (9H, -N$^+$-(C**H$_3$**)$_3$, 1.98-1.82 (6H, -(C**H$_3$)$_2$**Br initiating moiety), 1.4-0.51 (3H, -C**H$_3$** backbone).

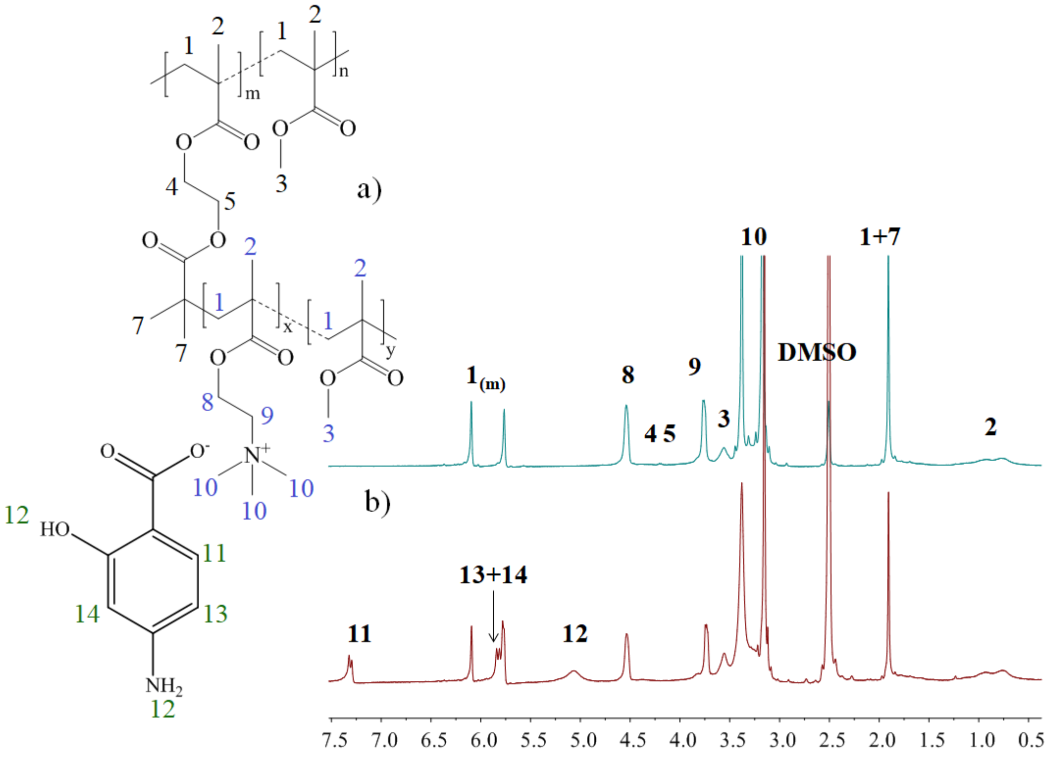


**Figure S4.** $^{1}$H NMR spectra of grafted copolymers a) G2, and b) G2_PAS, where * $\mathbf{1}_{(m)}$ is related to monomer residue.

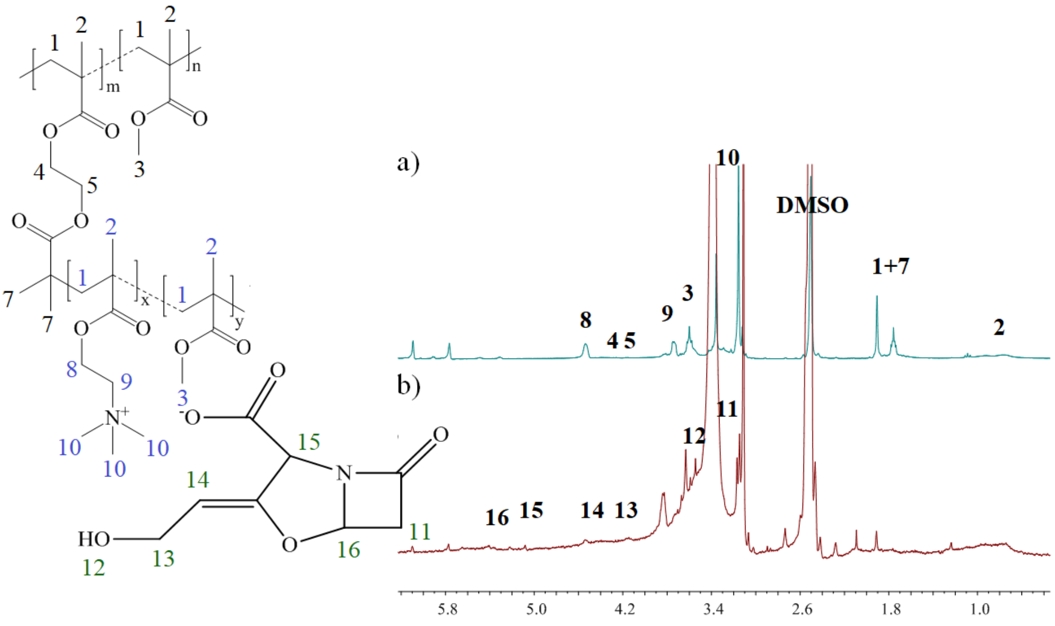


**Figure S5. .** $^{1}$H NMR spectra of grafted copolymers a) G5, and b) G5_CLV, where * $\mathbf{1}_{(m)}$ is related to monomer residue.

**Table S2.** Hydrodynamic diameters of nanoparticles determined with DLS[a].

| | Cl⁻ | | | PAS⁻ | | | CLV⁻ | | |
|---|---|---|---|---|---|---|---|---|---|
| | PDI | Size [nm] | Intensity [%] | PDI | Size [nm] | Intensity | PDI | Size [nm] | Intensity |
| **G1** | 0.361 | 88<br>368<br>21 | 56<br>25<br>17 | 0.503 | 145<br>23 | 59<br>40 | 0.601 | 148<br>18 | 75<br>25 |
| **G2** | 0.427 | 124<br>21 | 60<br>40 | 0.114 | 261 | 100 | 0.365 | 21<br>357 | 80<br>20 |
| **G3** | 0.295 | 203 | 98 | 0.775 | 354 | 100 | 0.442 | 26<br>250 | 69<br>31 |
| **G4** | 0.454 | 18<br>125 | 64<br>32 | 0.264 | 24 | 89 | 0.397 | 25<br>240 | 83<br>11 |
| **G5** | 0.444 | 114 | 94 | 0.265 | 75 | 100 | 0.268 | 91 | 100 |
| **G6** | 0.293 | 105 | 99 | 0.291 | 127 | 100 | 0.253 | 86 | 100 |
| **G7** | 0.241 | 72 | 100 | 0.443 | 194<br>37 | 82<br>18 | 0.316 | 75 | 98 |
| **G8** | 0.349 | 95 | 98 | 0.459 | 206<br>40 | 54<br>46 | 0.163 | 48 | 98 |

[a]concentration of copolymer in water: 1mg/mL.

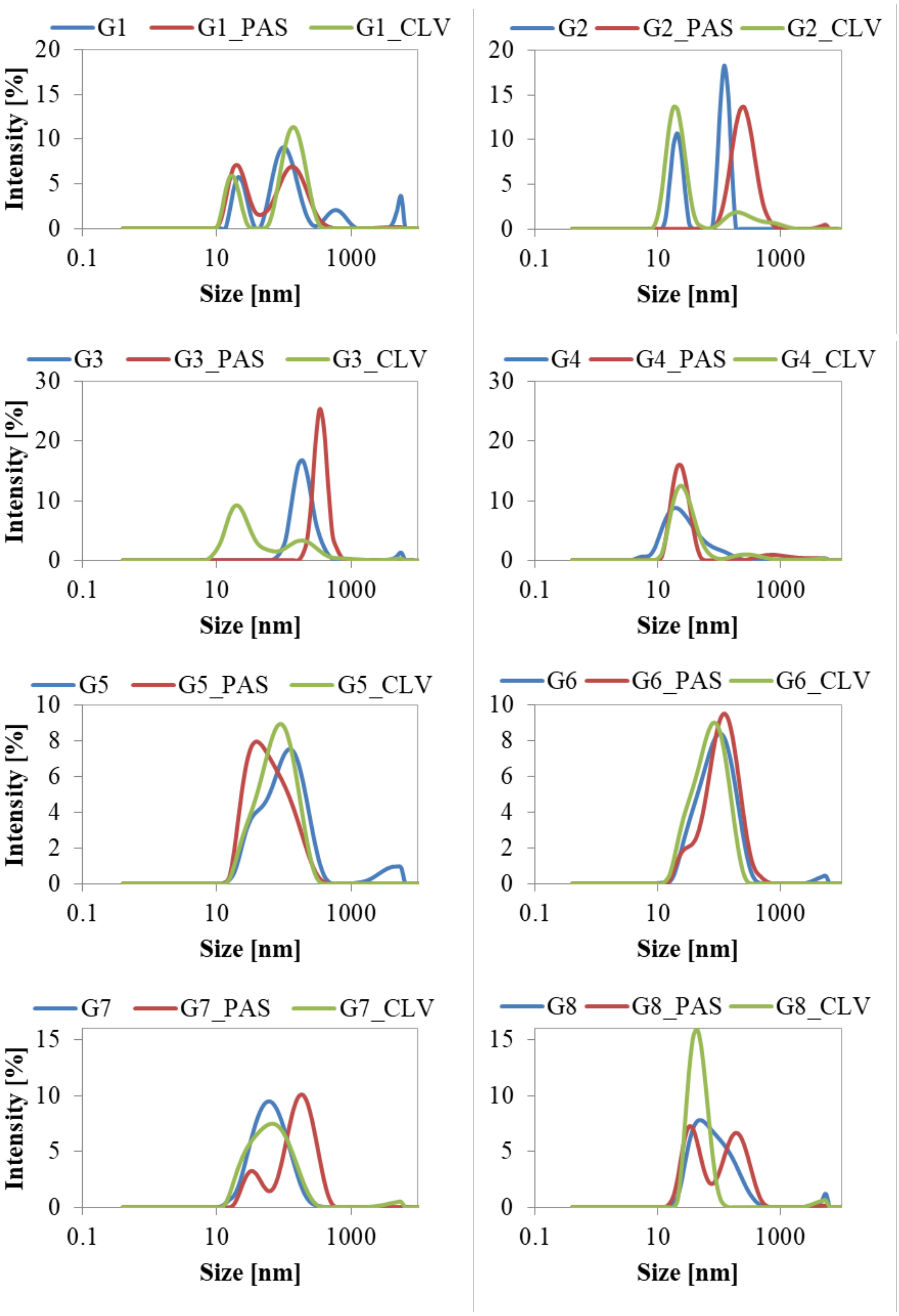


**Figure S6.** DLS histograms of particles formed by graft copolymers (1 mg/mL aq. solution).

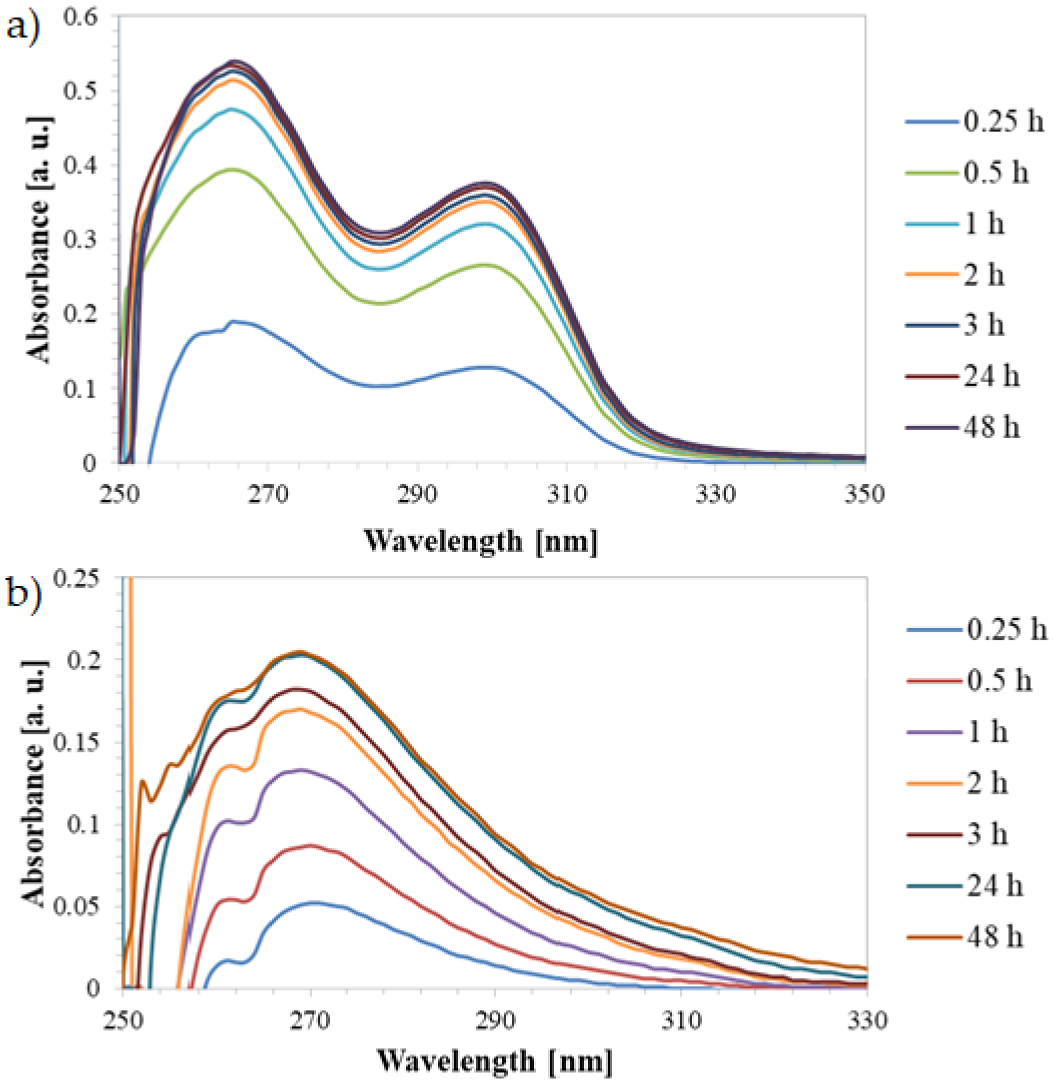


**Figure S7.** UV-Vis spectra of released drug for a) G2_PAS, and b) G2_CLV systems.
